# Dominant mutations in *CHK1* cause pronuclear fusion failure and zygote arrest that can be rescued by CHK1 inhibitor

**DOI:** 10.1038/s41422-021-00507-8

**Published:** 2021-05-06

**Authors:** Honghui Zhang, Tailai Chen, Keliang Wu, Zhenzhen Hou, Shigang Zhao, Chuanxin Zhang, Yuan Gao, Ming Gao, Zi-Jiang Chen, Han Zhao

**Affiliations:** 1 Center for Reproductive Medicine, Cheeloo College of Medicine, Shandong University, Jinan, Shandong China; 2 National Research Center for Assisted Reproductive Technology and Reproductive Genetics, Shandong University, Jinan, Shandong China; 3 Key laboratory of Reproductive Endocrinology of Ministry of Education, Shandong University, Jinan, Shandong China; 4 Shandong Provincial Clinical Medicine Research Center for Reproductive Health, Shandong University, Jinan, Shandong China; 5 Shanghai Key Laboratory for Assisted Reproduction and Reproductive Genetics, Shanghai, China; 6 Center for Reproductive Medicine, Ren Ji Hospital, School of Medicine, Shanghai Jiao Tong University, Shanghai, China

**Keywords:** Developmental biology, Mechanisms of disease, Embryonic stem cells

Dear Editor,

Infertility poses a major challenge to human reproductive health, affecting approximately 48 million women worldwide.^1^ Assisted reproductive techniques (ART), including in vitro fertilization (IVF) and intracytoplasmic sperm injection (ICSI), enable infertile women to have their biological embryos in vitro and further give birth to babies after embryo transfer. However, about 10% of all human embryos produced by ART are blocked in the early embryonic stage^2^ and approximately 2% fertilized oocytes derived from ART could not accomplish the first cell division.^3^ About one half of human infertility cases involve an underlying genetic factor, although the majority of genetic causes have remained elusive.^4^ In this study, we report that dominant mutations in *CHK1* are responsible for pronuclear fusion failure and zygote arrest (PFF-ZA) in 7 out of 29 cases, likely through increasing the CHK1 activity. Importantly, PFF-ZA caused by these mutations could be effectively rescued by using the CHK1 inhibitor, PF477736.

To uncover the genetic etiology of human zygote arrest, whole exome sequencing and Sanger sequencing analyses of 29 patients with zygote arrest were performed. We found seven patients in four independent families carrying heterozygous *CHK1* mutations (Family 1: c.1136 G>A, p.R379Q; Family 2: c.1323delC, p.F441fs*16; Family 3: c.1325 G>A, p.R442Q; Family 4: c.1259 G>A, p.R420K) (Fig. 1a), of which the amino acid residues (R379, F441, R442, R420) are conserved among different species (Supplementary information, Fig. S1). These patients underwent at least two failed IVF or ICSI attempts with arrested zygotes accompanying pronuclear fusion failure (Supplementary information, Table S1). Notably, in Family 1 the mutation c.1136 G>A, p.R379Q caused female infertility while the male carriers were unaffected, indicating an autosomal dominant inheritance pattern by paternal transmission (Fig. 1a). The four mutations were not found in either the standard public databases or among 300 fertile female controls. Furthermore, all of these mutations were classified as Likely Pathogenic according to the criteria of the American College of Medical Genetics and Genomics (ACMG) (Supplementary information, Table S2).

**Fig. 1:**
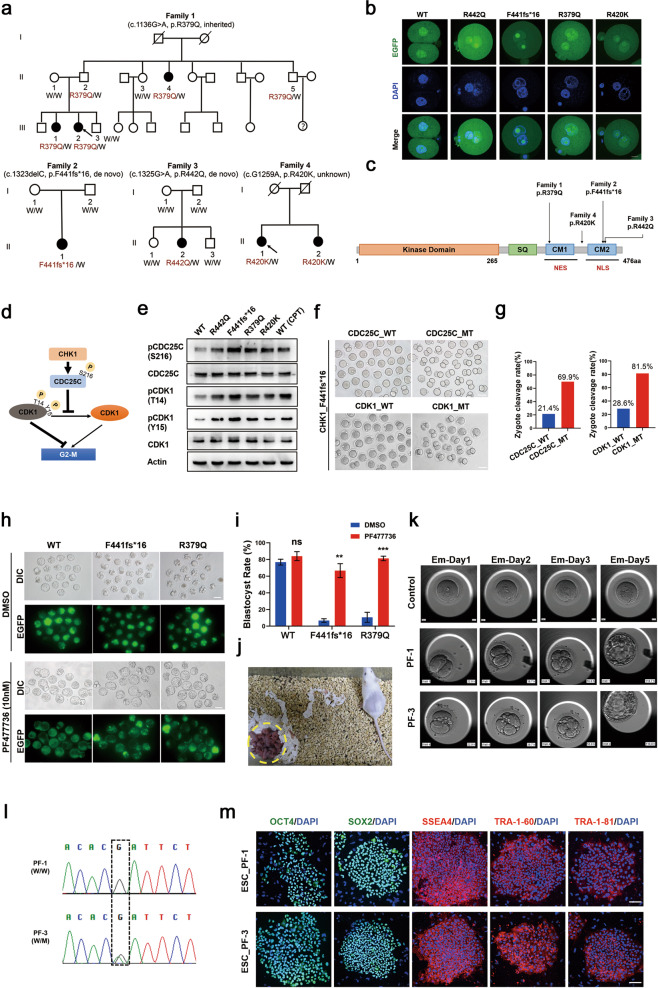
C-terminal mutations of *CHK1* cause pronuclear fusion failure and zygote arrest that can be rescued by CHK1 inhibitor. **a** Pedigrees of four families with CHK1 mutations. The squares denote male family members; circles represent female family members; solid symbols represent affected subjects; open symbols represent unaffected ones; slashes indicate death; question marks unknown fertility status; and arrows indicate probands. W, wild-type. **b** Fertilized eggs of mice were injected with either WT or mutant *hCHK1* cRNAs and then cultured in vitro for 18 h prior to fixation for immunofluorescence. Green, EGFP-tagged WT or mutant CHK1; blue, DAPI. Scale bar, 10 μm. **c** Schematic diagram of CHK1 protein showing its kinase domain, the C-terminal domain with SQ, CM1 and CM2 motifs, and the positions of altered amino acids. NES, nuclear export signal; NLS, nuclear localization signal. **d** A diagram showing the downstream pathway of CHK1 after activation. **e** HEK-293T cells were transfected with either WT or mutant hCHK1 constructs for 48 h in order to detect downstream CHK1 proteins. The WT group treated with camptothecin to induce DNA damage served as a positive control. **f**, **g** S216 in CDC25C and T14/Y15 in CDK1 were mutated to alanines. The CDC25C and CDK1 mutants were then respectively overexpressed in fertilized mouse eggs together with the CHK1 mutant F441fs*16. **f** Representative images revealing zygote cleavage in each group. WT, wild-type; MT, mutant. Scale bar, 100 μm. **g** Cleavage rate significantly increased in zygotes carrying mutated forms of CDC25C and CDK1 (*P* < 0.05; *χ2* test). The total cleavage rates of three replicates (about 80 eggs) are shown above the column. **h**, **i** Mouse zygotes overexpressing either WT or mutant CHK1 (F441fs*16 or R379Q) were cultured with or without PF477736 (10 nM). Representative images showing embryo development in each group (**h**). Scale bar, 100 μm. PF477736 markedly increased blastocyst rates in mutated groups (**i**, based on an unpaired *t*-test). Data are presented as means ± SEM; ns, no significant difference. **P < 0.01; ***P < 0.001 **j** Representative image of the offspring (yellow dotted circle) in a mutant group (R379Q). **k** Time-lapse imaging showing development progress of the embryo in control group and PF477736 treatment groups (PF-1 and PF-3). Em, embryo. **l** Chromatograms of Sanger sequencing of embryo PF-1 and PF-3. W, wild-type, M, mutant. **m** Expression of human ESC markers in the two embryo stem cell lines derived from PF-1 and PF-3, including OCT4, SOX2, SSEA4, TRA-1-60 and TRA-1-81. Scale bars, 100 μm.

*CHK1* encodes a serine/threonine-protein kinase which is required for checkpoint-mediated cell cycle arrest. To investigate its roles during early embryonic stage, we analyzed the expression pattern and found that both mRNA and protein levels of mouse CHK1 were relatively high in the zygote and two-cell embryo stages (Supplementary information, Fig. S2a–c), consistent with reported RNA-seq data of human early embryos^5^ (Supplementary information, Fig. S2d), suggesting that CHK1 may be a maternal factor that plays an important role in the process of fertilization. To validate that these mutations indeed cause PFF-ZA, we injected wild-type (WT) and mutant variants of human *CHK1* cRNA respectively into mouse zygotes with two distinct pronuclei (Supplementary information, Fig. S2g). The results showed that the mutant hCHK1 significantly decreased cleavage rate compared with WT hCHK1 (Supplementary information, Fig. S2e, f). Interestingly, the male and female pronuclei of zygotes injected with the mutant groups did not fuse, completely duplicating the phenotypes of PFF-ZA observed in the infertile patients, while zygotes injected with WT hCHK1 successfully developed to 2-cell stage at 18 h after injection (Fig. 1b). Taken together, these results strongly suggest that *CHK1* mutations cause PFF-ZA.

The CHK1 N-terminus has an extremely conserved kinase domain, while the C-terminus harbors a regulatory domain containing a Ser/Thr (SQ) motif and two highly conserved motifs (CM1 and CM2) (Fig. 1c). Interestingly, the four mutant amino acid residues at R379, F441, R442 and R420 are in or near the two conserved motifs (Fig. 1c). To investigate whether the conserved mutations can influence the structure of the protein, we analyzed its structure according to the three-dimensional structure prediction and found that R379 could form hydrogen bond with a nearby residue, while the hydrogen bond disappears after replacement of R379 with Q379 (Supplementary information, Fig. S3a). R442 can form four hydrogen bonds with the surrounding residues while the hydrogen bonds between R442 and the two residues (Y86 and C87) in the N-terminal domain disappear after R442 being replaced by Q442, accompanied by formation of a new hydrogen bond with L443 (Supplementary information, Fig. S3a). The substitution of residue R by Q also caused a change in the surface potential of the protein (Supplementary information, Fig. S3b), which might further affect function of the protein.

CHK1 mainly localizes to chromatin in nuclei in undisturbed condition; when activated, a proportion of the protein will be exported to the cytoplasm.^6^ *CHK1* mutations altered the protein structure and may further change its location. To investigate the localization patterns of mutant CHK1, we performed immunofluorescence staining and found that all of the protein variants exhibited increased cytoplasmic signals, especially the truncation mutant F441fs*16 which was almost completely absent from the nucleus (Supplementary information, Fig. S4). Given that nuclear export of the CHK1 protein is regulated by Chromosome maintenance protein 1 (Crm1) which binds to the nuclear export signal (NES, corresponding to CM1 domain) of the substrate,^7^ we thus used Leptomycin B (LMB), a Crm1-dependent NES inhibitor, to treat cultured cells and found that LMB abolished cytoplasmic localization of the R379Q, R442Q, and R420K mutants (Supplementary information, Fig. S5), demonstrating that the NES plays an important role in the inclination of cytoplasmic localization for these mutants. However, the mutant F441fs*16 still showed cytoplasmic localization after LMB treatment (Supplementary information, Fig. S5), indicating that cytoplasmic localization of the truncated protein was the result of nuclear localization signal (NLS, corresponding to CM2 domain) disruption.

CHK1 holds a closed conformation by the interaction between N-terminal kinase domain and C-terminal regulatory domain; while mutations in conserved motifs CM1 or CM2 could destroy this closed conformation, exposing its kinase domain and thus activating CHK1.^8^ Indeed, analysis of kinase activity showed that CHK1 mutants exhibited increased kinase activities compared to WT (Supplementary information, Fig. S6). Activated CHK1 can directly phosphorylate CDC25C at S216, resulting in the accumulation of inhibitory CDK1 phosphorylated at both T14 and Y15, thus preventing the G2/M transition and causing cell cycle arrest (Fig. 1d).^9^ Moreover, the late pronucleus stage of the zygote corresponds to the G2 stage, after which the zygote enters mitosis.^10^ We therefore detected the phosphorylation levels of downstream CHK1 effectors regulating G2/M transition in HEK-293T cells carrying mutant forms of CHK1 and found increased accumulation of phosphorylated CDC25C (S216) and CDK1 (T14 and Y15), similar to results obtained by treatment with CPT (a DNA damage drug that activates CHK1), while the truncation mutation led to the highest accumulation of inhibitory pCDK1 (Fig. 1e). To further validate the downstream targets of mutant CHK1 in fertilized eggs, we expressed mutant CHK1 harboring F441 fs*16 in mouse zygotes, along with mutated CDC25C or CDK1 lacking their phosphorylation sites. Our results showed that both mutated CDC25C and CDK1 were able to overcome the F441fs*16-induced zygote arrest (Fig. 1f, g). Taken together, these results suggest that *CHK1* mutations increase the kinase activity of CHK1 in zygotes and cause PFF-ZA through accumulation of inhibitory CDK1 that induces zygote G2/M transition arrest.

We next examined whether inhibition of kinase activity of CHK1 can rescue the zygote arrest phenotype. We employed a CHK1 inhibitor PF477736, which has been previously applied to inhibit CHK1 activity in a phase I clinical trial of a combined tumor treatment with gemcitabine.^11^ We first optimized the concentration of PF477736 and found that inhibitor at 10 nM had the best efficiency to increase the blastocyst yields in mouse mutant groups (Fig. 1h, i; Supplementary information, Fig. S7). Copy number variant (CNV) analysis indicated that these blastocysts sustained genetic integrity (Supplementary information, Fig. S8 and Table S3). In addition, PF477736 could also markedly increase the rates of zygote cleavage (Supplementary information, Fig. S9a, b) probably by decreasing the levels of pCDC25C and pCDK1 in all mutant groups (Supplementary information, Fig. S9c). Strikingly, in vivo developmental experiment (Supplementary information, Fig. S10a) showed that the treated embryos gave rise to healthy pups (Fig. 1j) with similar birth rate or body weight (till 12 weeks) compared to those of controls (Supplementary information, Fig. S10b, c).

In order to further evaluate efficacy and safety of the CHK1 inhibitor, we treated the donated frozen zygotes from patient III-2 (Family 1) with PF477736, which had been extendedly cultured until the third day after fertilization without division. Surprisingly, these blocked zygotes were able to divide and recover mitosis (Supplementary information, Fig. S11). Subsequently, in another oocyte-retrieval cycle, we treated five fresh fertilized eggs donated by the same patient with PF477736 right after the formation of pronuclei. Amazingly, we observed that while the two untreated control zygotes still stayed in the pronuclei stage and never divided as expected, all five zygotes treated with PF477736 overcame one cell stage and two of them even developed into good-quality blastocysts (PF-1 and PF-3) (Fig. 1k; Supplementary information, Video S1). Genotyping analysis showed one blastocyst was WT while the other carried R379Q mutation (Fig. 1l). Furthermore, both of the two blastocysts were successfully derived into human embryonic stem cells (ESC_PF-1 and ESC_PF-3) exhibiting pluripotency (Fig. 1m) and genetic testing proved their genetic integrity (Supplementary information, Fig. S12). Taken together, these results demonstrate that mutant CHK1 protein with increased kinase activity in oocytes induces division failure of zygotes; inhibiting CHK1 kinase activity could successfully recover zygote division and accomplish the transition from meiosis to mitosis in early embryo development.

In conclusion, we have identified novel dominant genetic mutations in *CHK1* that cause female infertility induced by zygote arrest, characterized by pronuclear fusion failure. We have also demonstrated that increased CHK1 activity caused by mutations arrests G2/M transition of zygotes. Importantly, the usage of the inhibitor of CHK1 to suppress its kinase activity can rescue the zygote arrest phenotype in both mouse and human, offering an effective and safe intervention for the treatment of this type of infertility.

## Supplementary information

Supplementary Figures and tables

Supplementary Video 1
