# Supplementary material for: Dominant mutations in CHK1 cause pronuclear fusion failure and zygote arrest that can be rescued by CHK1 inhibitor
Source: Cell Res. 2021 May 6;31(7):814–7. doi: 10.1038/s41422-021-00507-8 (PMC8249641; doi:10.1038/s41422-021-00507-8)
Supplement: Supplementary file 1 — Supplementary Figures and tables [file 41422_2021_507_MOESM1_ESM.pdf]

SUPPLEMENTARY INFORMATION

Dominant mutations in *CHK1* cause pronuclear fusion failure and zygote arrest that can be rescued by CHK1 inhibitor

Honghui Zhang[1,2,3,4], Tailai Chen[5,6], Keliang Wu[1,2,3,4], Zhenzhen Hou[1,2,3,4], Shigang Zhao[1,2,3,4], Chuanxin Zhang[1,2,3,4], Yuan Gao[1,2,3,4], Ming Gao[1,2,3,4], Zi-Jiang Chen[1,2,3,4,5,6], Han Zhao[1,2,3,4]

[1]Center for Reproductive Medicine, Cheeloo College of Medicine, Shandong University, Jinan, Shandong, 250012, China;

[2]National Research Center for Assisted Reproductive Technology and Reproductive Genetics, Shandong University, Jinan, Shandong, 250012, China;

[3]Key laboratory of Reproductive Endocrinology of Ministry of Education, Shandong University, Jinan, Shandong, 250012, China;

[4]Shandong Provincial Clinical Medicine Research Center for Reproductive Health, Shandong University, Jinan, Shandong, 250012, China;

[5]Shanghai Key Laboratory for Assisted Reproduction and Reproductive Genetics， Shanghai, 200000, China;

[6]Center for Reproductive Medicine, Ren Ji Hospital, School of Medicine, Shanghai Jiao Tong University, Shanghai, 200000, China

These authors contributed equally: Honghui Zhang, Tailai Chen, Keliang Wu, Zhenzhen Hou

Correspondence: Zi-Jiang Chen (chenzijiang@hotmail.com) or Han Zhao (hanzh80@sdu.edu.cn)

LIST OF CONTENTS

Materials and Methods 3

- **Human subjects** 3
- **The CHK1 inhibitor (PF477736) treatment and embryo transfer** 5
- **Whole exome sequencing, data analysis and validation** 6
- **Expression construction** 7
- **Mouse oocytes/embryos collection** 7
- **Quantitative RT-PCR** 8
- **In vitro cRNAs synthesis and microinjection** 8
- **Embryo immunofluorescence** 9
- **Molecular modeling and evolutionary conservation analysis** 9
- **Cell transfection and immunofluorescence** 9
- **CHK1 kinase activity assay** 10
- **Western blot** 10
- **Copy number variant (CNV) analysis** 11
- **Derivation of human embryonic stem cell (hESC) lines** 11
- **Statistics analysis** 12

Supplementary Figures 13

- **Figure S1. Sanger sequencing chromatograms of the four families and conservation analysis of the mutated amino acid residues.** 13
- **Figure S2. The mutations in *CHK1* could cause mouse zygote cleavage failure.** 14
- **Figure S3: Structural exhibition of CHK1.** 16
- **Figure S4: The changed localization of mutations in HEK-293 cells** 18
- **Figure S5: The dis-localization of mutants in HEK-293 cells is induced by NES or NLS.** 19
- **Figure S6: The CHK1 mutants hold increased kinase activities.** 20
- **Figure S7: Optimizing the concentration of PF477736** 21
- **Figure S8: Representative CNV-seq images of mouse blastocysts treated with PF477736.** 22
- **Figure S9. The CHK1 inhibitor can rescue mouse zygote arrest.** 23
- **Figure S10: Mouse mutant zygotes can generate normal offspring under the treatment with PF477736.** 25
- **Figure S11. The blocked zygotes from patient III-2 (Family 1) could resume cleavage under the treatment with PF477736** 26
- **Figure S12. CNV-seq results of the two embryonic stem cell lines derived from patient (III-2, Family 1).** 27

Supplementary Tables 28

- **Table S1. Oocyte and embryo characteristics of IVF and ICSI cycles in the patients with *CHK1* mutations.** 28

Table S2: Overview of the *CHK1* mutations observed in the four families 29
Table S3: CNV-seq results of mouse blastocysts with mutations after treatment with PF477736 30
Table S4: Mouse embryo transfer record. 31
Table S5: Embryo development record after treatment with CHK1 inhibitor PF477736. 32
Table S6: Primers used for *CHK1* exon sequencing 33
Table S7: Primers used for site-directed mutagenesis and qRT-PCR 34
Table S8: List of antibodies used in immunofluorescence and western blot 35
Supplementary References 36
Web Resources 37

Materials and Methods

Human subjects

Patients with familial or sporadic zygote arrest, as well as healthy control individuals were recruited in the Center for Reproductive Medicine, Shandong University, China. All subjects signed informed consents, and this study was reviewed and approved by the Institutional Review Board of Reproductive Medicine, Shandong University.

The proband (III-2) in Family 1 was 28 years old and had been infertile for 3.5 years without contraception. She had regular menstrual cycles with normal sex hormone level, and the sperm count, morphology and motility of her spouse were normal too. She was diagnosed as primary infertility. Three IVF/ICSI cycles were performed and a total of 24 fertilized eggs were obtained. However, the majority of the zygotes arrested in the pronuclei (PN) or 1-cell stages on the first day after fertilization when the embryos from normal controls are usually in 2-cell stage. Almost none of them divided in the

next three days, resulting in no transferable embryos. We regard this phenotype as zygote arrest chiefly characterized by pronuclei fusion failure (PFF-ZA). What's more, it is worth noting that an elder sister and an aunt of the patient also suffered from infertility.

The patient (II-1) in Family 2 was 31 years old. Although the menstrual cycle and sex hormone levels were normal, she had a 7-year history of primary infertility. Then she tried three IVF/ICSI cycles in our center, and a total of 25 fertilized eggs were obtained. On the first day of cleavage, 23 fertilized eggs still had PN and only 2 eggs were in 1-cell stage. Almost all of them did not divide and still showed clear PN in the next few days, which was much more serious than the condition of patient in Family 1.

The third patient (II-2) we found in Family 3 was 27 years old. Similar to the former two patients, she had a 5-year history of primary infertility with regular menstrual cycle and normal sex hormones. She had four IVF/ICSI cycles and obtained a total of 26 fertilized eggs, of which 23 fertilized eggs were blocked at PN stage and only 2 eggs in 1-cell stage on the first day of cleavage. No transferable embryo could be used either.

The proband (II-1) in Family 4, 36 years old, had a 7-year history of infertility with normal menstrual cycle and sex hormone levels, diagnosed as primary infertility. She performed two IVF/ICSI cycles and a total of 10 fertilized eggs were obtained, among which five were in PN in the first cleavage day and the others were in 1-cell stage. Most of the embryos were not divided and there were no transferable embryos as well. The

younger sister of the patient with a 10-year history of infertility had two failed IVF/ICSI treatments and also showed zygote arrest.

The CHK1 inhibitor (PF477736) treatment and embryo transfer

The mouse zygotes overexpressing the mutation p.F441fs*16 or p.R379Q were treated with PF477736 at 1nM, 10nM or 100nM until the 2-cell embryo stage, and the embryos were then transferred to a medium without PF477736 for culturing until the blastocyst stage.

For mouse embryo transfer experiment, WT or mutant human *CHK1* cRNA was injected into zygotes from C57 mouse (Beijing Vital River Laboratory Animal Technology Co.). Those fertilized eggs were then cultured until 2-cell embryo stage in M16 medium (Sigma, M7292) containing 10nM PF477736 (Selleck, S2904), transplanted into pseudo-pregnant ICR female mice together with control embryos, and then the litter sizes and body weight of each group were observed. See embryo transfer record in Supplementary information, Table S4.

Donated frozen zygotes or fresh zygotes from the patient III-2 in Family 1 were treated with10nM PF477736 until the 2-cell embryo stage, and the embryos were immediately transferred to a medium without PF477736 for culturing until the blastocyst stage. See embryo development record in Supplementary information, Table S5.

Whole exome sequencing, data analysis and validation

The DNA of human peripheral blood was extracted by QIAamp DNA Mini Kit according to the manufacturer's instruction. Exome capture and sequencing were performed using Agilent SureSelect Whole Exome capture and Illumina platform. A variant was considered to be a candidate mutation if it 1) had not been reported previously or had a prevalence below 0.01% in the three public databases (dbSNP, 1000 Genome, and gnomAD); 2) was a non-synonymous SNP/insertion/deletion in the coding region or in splicing region; 3) was predicted to be harmful via at least two software, such as SIFT, Polyphen-2 and Mutation Taster. Next, the filtered candidate mutations were verified by sanger sequencing in the family members, excluding ones that were not co-segregated with the disease. Finally, the candidate mutations were further verified in 300 fertile women in our center to remove the loci in normal controls. See the primers in supplementary information, Table S6.

We found seven patients in four independent families carrying heterozygous *CHK1* mutations (Family 1: c.1136G>A, p.R379Q, inherited; Family 2: c.1323delC, p. F441fs*16, de novo; Family 3: c.1325 G>A, p.R442Q, de novo; Family 4: c.1259 G>A, p. R420K, unknown). Haplotype analysis proved the paternity relationship between the patient and their parents in Family 2 and Family 3. In addition, no matter the zygotes carried the CHK1 mutation or not, almost all the patients' fertilized eggs were arrested at zygote stage and never divided, indicating maternal factor may contribute to the phenotype.

Expression construction

Primers were designed to amplify the target gene from pENTER vector (Vigene Biosciences) containing the full-length coding sequence of human *CHK1* (NM_001274). Then the *CHK1* gene was cloned into the pcDNA3.1 (+) vector together with the enhanced green fluorescent protein (EGFP) or red fluorescent protein (mCherry) coding sequence, in order to obtain the CHK1 fusion protein with green or red fluorescent protein tag at the N-terminus. According to the manufacturer's method, the plasmid containing the coding sequence of EGFP and CHK1 was mutagenized by Quick Change Lightning Site-Directed Mutagenesis Kit (Agilent Technologies) to obtain CHK1 mutated plasmids (c.G1136A, c.1323delC c.G1325A, and c.G1259A). The mutant plasmids CDC25C (BC019089.2) and CDK1 (NM_001786.4) were obtained with the same kit. The primers for site-directed mutation can be found in Supplementary information, Table S7.

Mouse oocytes/embryos collection

The 6-8 weeks healthy ICR female mice (Beijing Vital River Laboratory Animal Technology Co.) were super-stimulated with 7.5 IU pregnant mare’s serum gonadotropin (PMSG, NINGBO SANSHENG) followed by 7.5 IU human chorionic gonadotropin (HCG, NINGBO SANSHENG) after 44-48h. We then collected cumulus oocyte complex (COC) in the ampulla of mouse oviduct 18 hours later. Sperm from the cauda epididymidis of 8-12 weeks ICR male mice (Beijing Vital River Laboratory Animal Technology Co.) were capacitated in G-IVF medium (Vitrolife) for 1 hour. Then

the harvested COC and capacitated sperm were added to new G-IVF medium covered with mineral oil for 4 to 6 hours at 37 ºC in a 5 % CO_2 atmosphere to obtain fertilized eggs, which would be transferred into KSOM medium (Sigma Aldrich) covered with mineral oil later to obtain 2-cell, 4-cell, 8-cell, morula and blastocyst stage embryos. GV oocytes were obtained from mouse ovaries 44 hours after PMSG injection. MII oocytes need to be digested with hyaluronidase (Sigma-Aldrich) to remove granulosa cells.

Quantitative RT-PCR

Mouse oocytes and embryos at different development stages were applied to obtain cDNA with REPLI-g WTA Single Cell Kit (QIAGEN) according to the manufacturer's instructions. Power SYBR Green Master Mix (Takara) was used for qRT-PCR analysis on Roche 480 PCR system. The relative expression level of CHK1 equals $1000 \cdot 2^{-\Delta Ct}$, of which Δ Ct=Ct (CHK1)-Ct (GAPDH). See the qRT-PCR primers in Supplementary information, Table S7.

In vitro cRNAs synthesis and microinjection

The plasmids were linearized with appropriate restriction endonuclease. According to the factory's method, 5' capped cRNAs were synthesized via mMESSAGE mMACHINE T7 Transcription Kit (Invitrogen, AM1344) and then added with poly (A) tail using Poly(A) Tailing Kit (Invitgen, AM1350), followed by purification with RNeasy

MinElute Cleanup Kit (QIAGEN,74204) and dilution in nuclease-free water. About 5pl cRNA solution (1400ng/ul) was microinjected into the cytoplasm of the fertilized eggs.

Embryo immunofluorescence

Mouse embryos were fixed in 4% paraformaldehyde (Solarbio) for 30 minutes and permeated in PBS containing 0.3% TritonX-100 for 20 min. After being blocked in 1% bovine serum albumin (BSA, Sigma) in PBS for 1 h, they would be re-stained with 4-methyl-6-methyl-2-phenylindole (DAPI, Vector Laboratories) for 10 minutes. After mounting, oocytes/embryos were examined with a confocal laser-scanning microscope (Dragonfly, Andor, England).

Molecular modeling and evolutionary conservation analysis

The three-dimensional structures of CHK1 (NP_001265.2) were predicted by SWISS-MODLE webserver (PDB ID:6C9D). Molecular graphics and analysis were carried out by PyMol software. Evolutionary conservative analysis was performed with Clustalx software.

Cell transfection and immunofluorescence

HEK-293(T) cells were cultured in DMEM/high glucose medium (HyClone, SH30243.01B) with 10% fetal bovine serum (FBS, BI, 04-001-1ACS) at 37 ºC with 5 % CO_2. When the cell density reached 70%-80% fusion, they would be transfected by Lipofectamine 3000 Transfection Kit (Invitrogen, L3000015) according to the scheme given by the manufacturer.

HEK-293 cells growing on glass slides (NEST,801007), co-transfected with mCherry-WT and EGFP-WT or mutated Chk1 for 48 hours, were rinsed with warm PBS followed by being fixed with 4% paraformaldehyde at room temperature for 20 minutes. After being washed 3 times with cold PBS, they would be permeabilized in PBS containing 0.3 % Triton X-100 for 20 min, blocked with 5 % BSA in PBS for 1 h, and then re-stained by DAPI for 10 minutes. For embryonic stem cells, they would be incubated with primary antibodies overnight at 4 ºC after blocking, followed by incubation of secondary antibodies (invitrogen) for 1 hour at room temperature. The antibodies are shown in Supplementary information, Table S8.

CHK1 kinase activity assay

HEK-293T cells transfected with WT or mutant CHK1 constructions were collected after 48 hours. The activity of CHK1 kinase in different groups was detected by 96-well Checkpoint Kinase Activity Assay Kit (STA-414, Cell Biolabs) according to the manufacturer's instructions. The relative kinase activity was expressed by the ratio of OD value (450nm) of all groups to OD value of WT group.

Western blot

100 oocytes/early embryos or collected HEK-293T cells were lysed in protein lysis buffer containing protein phosphatase inhibitor (Beyotime, P1046) for about 30 min, and then denatured for 10min at 95 °C. The proteins were separated by SDS-polyacrylamide gel electrophoresis (SDS-PAGE) and transferred to PVDF membrane

(Millipore). The primary antibodies were incubated overnight at 4 ºC, then the HRP-conjugated secondary antibodies were incubated at room temperature for 1 hour. The membranes were eventually developed by Image Lab gel imaging system (Bio-Rad). The antibodies used are shown in Supplementary information, Table S8.

For HEK-293T cells, they were transfected with WT or mutant CHK1 constructions and then collected after 48 hours, followed by the treatment of 500nM CPT (Sigma, C9911) for another 2 hours in one of the two WT groups.

Copy number variant (CNV) analysis

Whole genome amplification (WGA) was performed, according to manufacturer's instructions, using the SurePlex WGA (VeriSeq PGS Kit, Illumina). The high-throughput sequencing platform, DA8600, was used for sequencing. CNV analysis of mouse blastocysts was done by aligning the sequence of mutant blastocysts treated by PF477736 with the sequence of normal control blastocysts to detect if there are chromosome aneuploidy abnormalities or chromosomal deletions or duplications larger than 4Mb. For ESCs derived from embryos treated with PF477736, WGA was performed with the same method using one outgrowth of the cell lines, followed by library preparation and sequencing on the Miseq system (Illumina).

Derivation of human embryonic stem cell (hESC) lines

The inner cell mass (ICM) of the patient's blastocysts (PF-1 and PF-3) produced by treatment with PF477736 were planted on mitotically inactivated mouse embryonic

fibroblasts (MEF) in modified human embryonic stem cell culture medium[1] in a humidified incubator at 37°C, 6% CO_2 5% O_2. Culture medium was usually changed every day. Outgrowths were formed after five days and passaged on fresh MEF feeders followed by mechanically separating into several pieces.

Statistics analysis

GraphPad Prism 8.0 was used for statistical analysis. Most experiments were repeated at least three times. Unpaired t-test or chi-square test was used for the comparison between two groups. The significant evaluation style of GraphPad is as follows: **P<0.01, ***P<0.001, ****P<0.0001.

Supplementary Figures

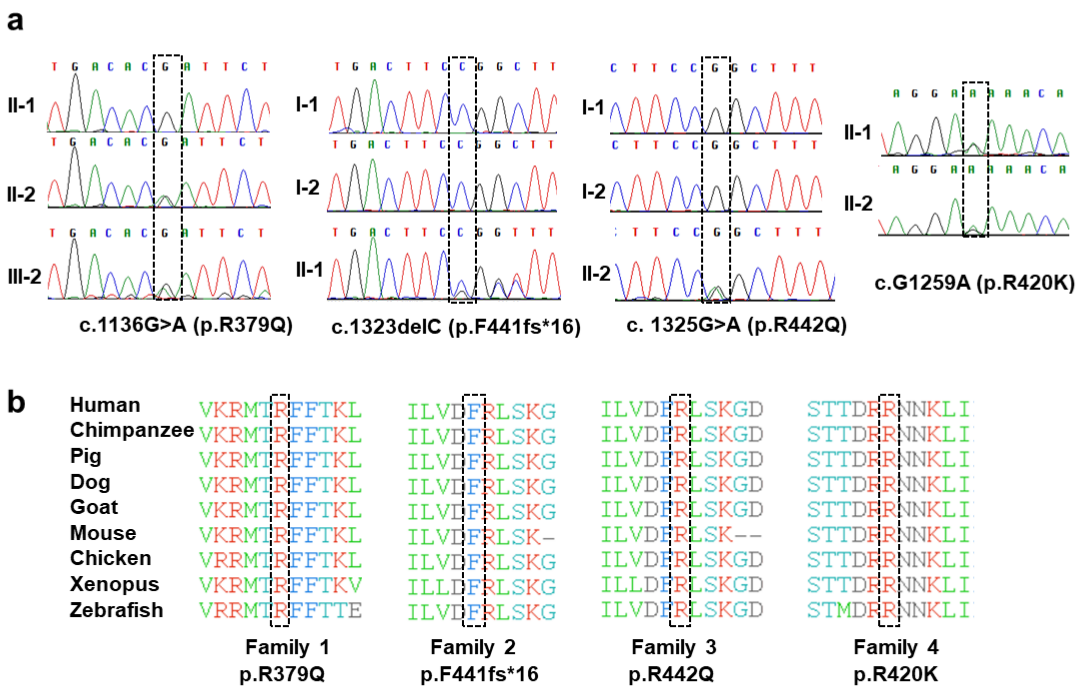

Figure S1. Sanger sequencing chromatograms of the four families and conservation analysis of the mutated amino acid residues.

a Chromatograms of Sanger sequence in the four independent families. **b** Sequence alignment reveals evolutionary conservation of amino acid residues R379 in Family 1, F441 in Family 2, R442 in Family 3 and R420 in Family 4 in nine species.

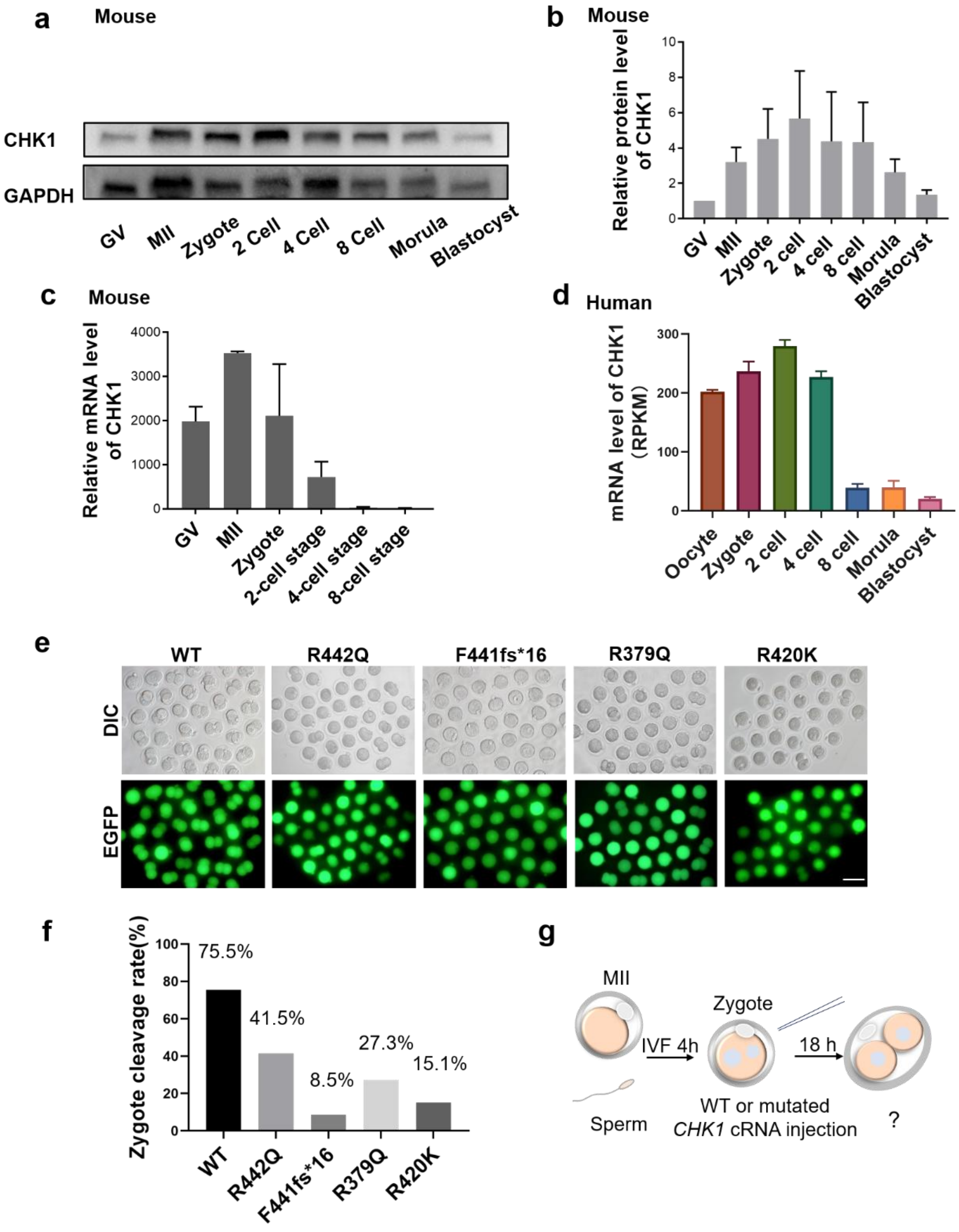

Figure S2. The mutations in *CHK1* could cause mouse zygote cleavage failure.

a Western blot result showed the expression of CHK1 in mouse oocytes and early embryos at different stages. GAPDH was used as a loading control. **b** Relative quantitative result of the protein expression of CHK1 in mouse oocytes and early embryos. **c** RT-PCR results show the mRNA level of CHK1 in mouse oocytes and early

embryos. **d** RNA-seq result of CHK1 in human mature oocytes and preimplantation embryos according to the published data[2]. Bars indicate means ± SEM. **e** Images showing mouse zygotes overexpressing WT or mutant human EGFP-CHK1 after 18 hours. Scale bar:100μm. **f** Zygote cleavage rate, shown as the overall rate across three experiments (about 90 eggs in each group), was significantly decreased in groups with mutated hCHK1 compared with WT group (P<0.05), based on the chi-square test. **g** A diagram depicting flow of mouse zygote injection. MII oocytes and capacitated spermatozoa were fertilized in vitro for 4 hours, then EGFP-tagged wild-type (WT) or mutant *hCHK1* cRNA was injected into the cytoplasm of fertilized eggs and subsequently cultured for 18 hours to observe the zygote cleavage rate.

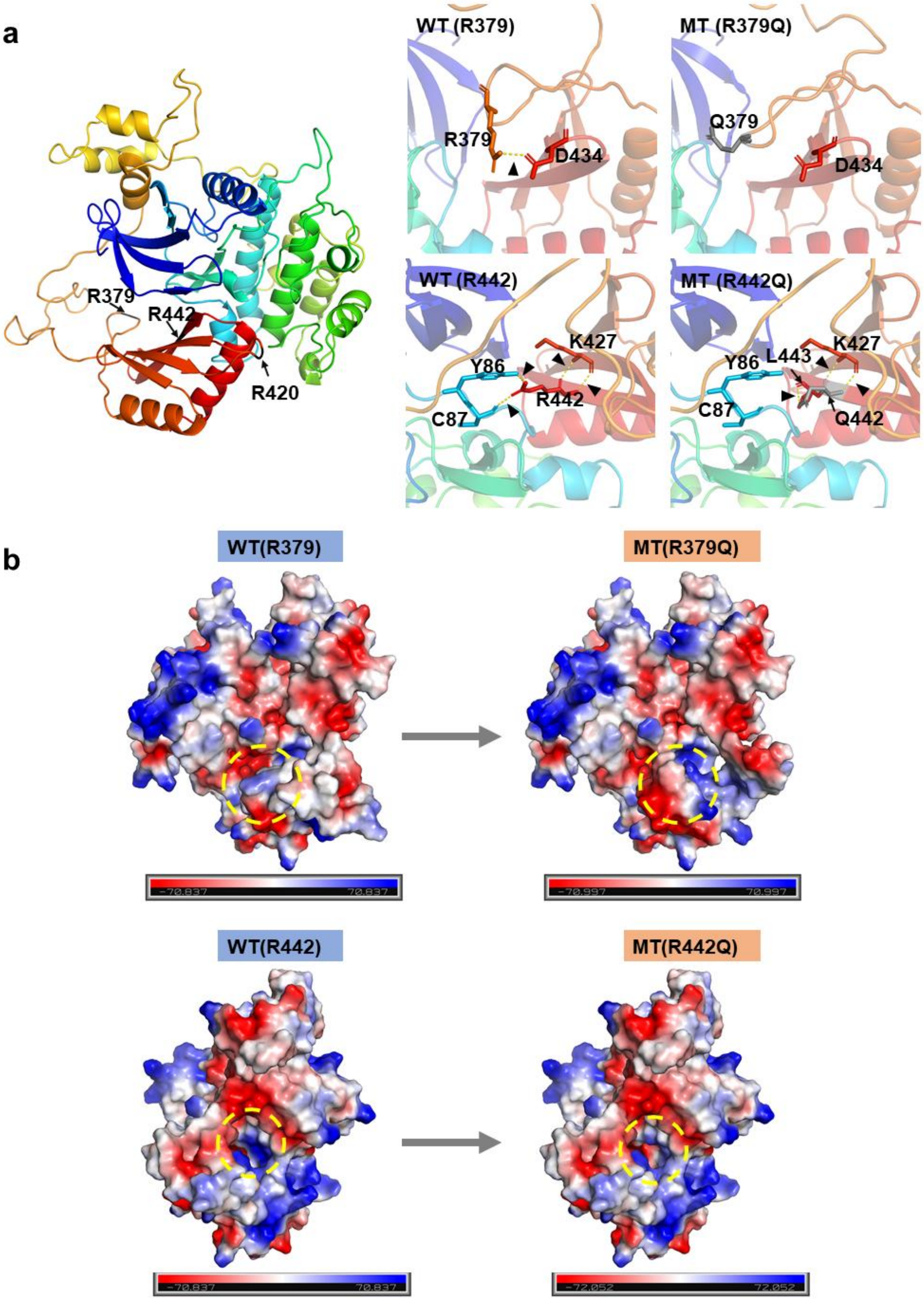

Figure S3: Structural exhibition of CHK1.

a Overview of the predicted structure of the wild-type (WT) CHK1 protein (Left). Arrow indicates the location of R379, R442 and R420. Magnified view of the predicted structures surrounding R379 and R442 and their altered structures after mutation (Right). R379 could form hydrogen bond with surrounding residue D432, while the

hydrogen bond disappears after replacement of Q379. R442 can form four hydrogen bonds with the surrounding residues while the hydrogen bonds between R442 and the two residues (Y86 and C87) in the N-terminal domain disappear after R442 being replaced by Q442, accompanied by forming a new hydrogen bond with L443. The yellow dashed lines indicated by black arrowheads represent the predicted hydrogen bonds. The arrows mark the mutated amino acid Q442 and the amino acid L443 with newly formed hydrogen bond. **b** Schematic illustrations show that the R379Q mutation and R442Q mutation convert positively charged patch to negatively charged patch (in yellow circle) on the surface of CHK1. Bars indicate the electrostatic property with blue representing positive charge and red representing negative charge.

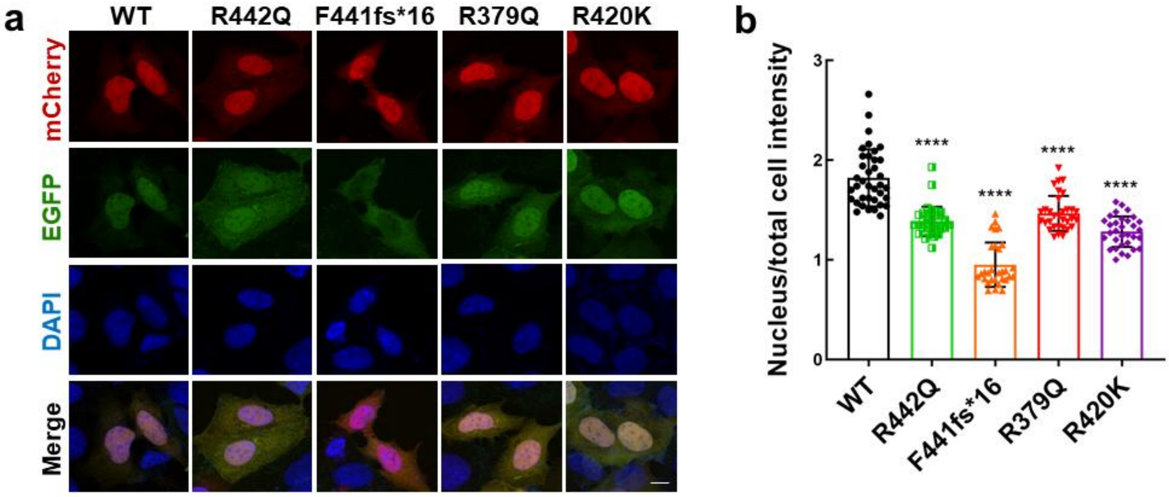

Figure S4: The changed localization of mutations in HEK-293 cells.

a Immunofluorescence results of HEK-293 cells co-transfected with mCherry-WT CHK1 and EGFP-CHK1 (WT or mutant) to show the intracellular localization of proteins. Red: mCherry-WT CHK1; Green: EGFP-WT or EGFP-mutated CHK1; Blue: DAPI; **b** Relative fluorescence intensity of the nucleus compared to the total cell of wild-type or mutant CHK1 in HEK-293 cells. Error bars, S.E.M. ****P < 0.0001 using two-tailed Student's t-tests.

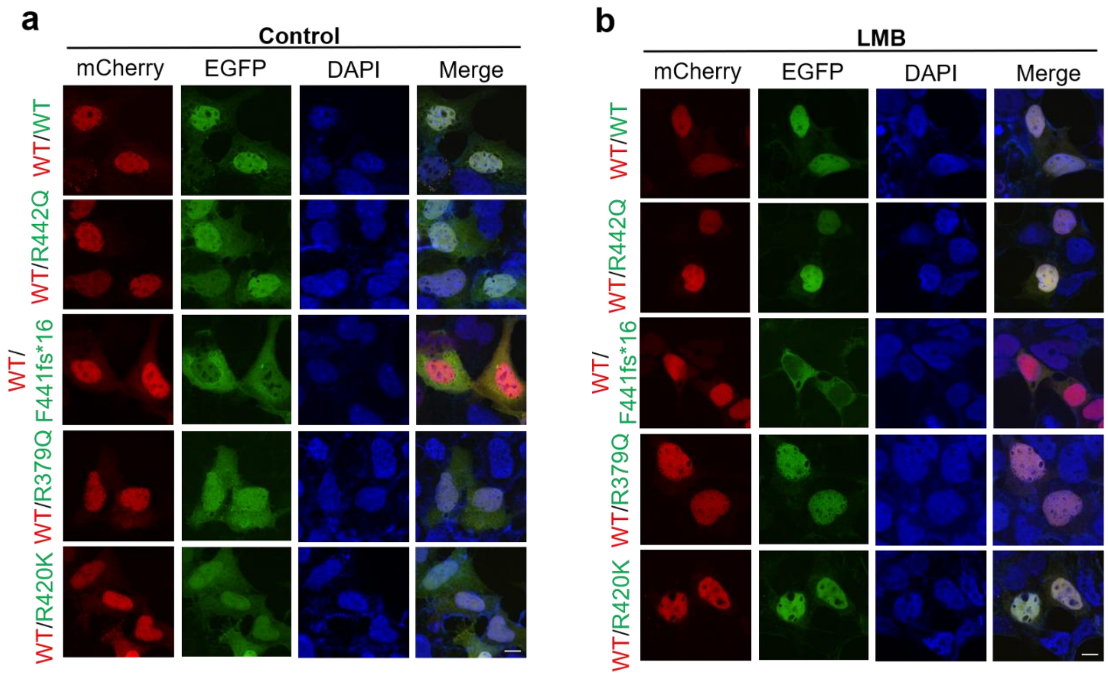

Figure S5: The dis-localization of mutants in HEK-293 cells is induced by NES or NLS.

a-b HEK-293 cells were treated with or without leptomycin B (LMB), a Crm1 inhibitor to inhibit nuclear export signal (NES)[3], for 15 h after transfection for 30 h. Red: mCherry-WT CHK1, Green: EGFP-WT or -mutated CHK1, Blue: DAPI. **a** The mutant groups, especially the F441fs*16 mutant, had a tendency of cytoplasmic localization compared with WT group **b** After LMB treatment, the mutant groups exhibit almost complete nuclear localization, except F441fs*16. Scale bar:10μm.

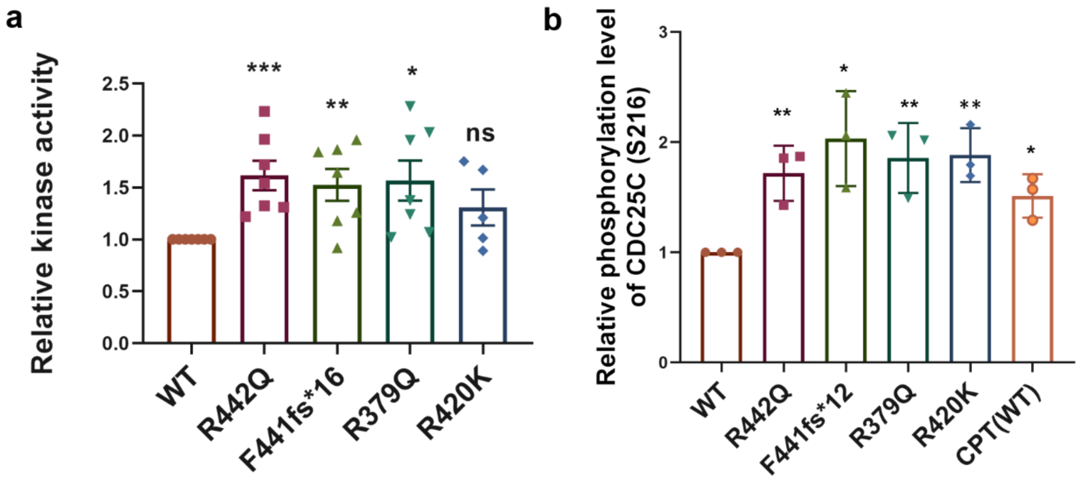

Figure S6: The CHK1 mutants hold increased kinase activities.

a HEK-293T cells were collected to assay kinase activity 48 hours after transfection with wild-type (WT) or mutant CHK1 plasmids. The results showed that the relative kinase activity (OD $_{\text{wt or mutant group}}$/ OD $_{\text{wt group}}$ in each replicate) of each mutation group was higher than that of the WT group, though the R420K mutation showed no significant difference (t-test). Bars indicate means ± SEM, ns: no significant difference. **b** It is known that CHK1 can directly phosphorylate CDC25C at Ser216. Here we also calculated the phosphorylation level of CDC25C (S216) in the manuscript (Fig. 1e) to evaluate the kinase activity of CHK1s (t-test). The result shows that the mutants all have increased kinase activity. Bars, S.E.M.

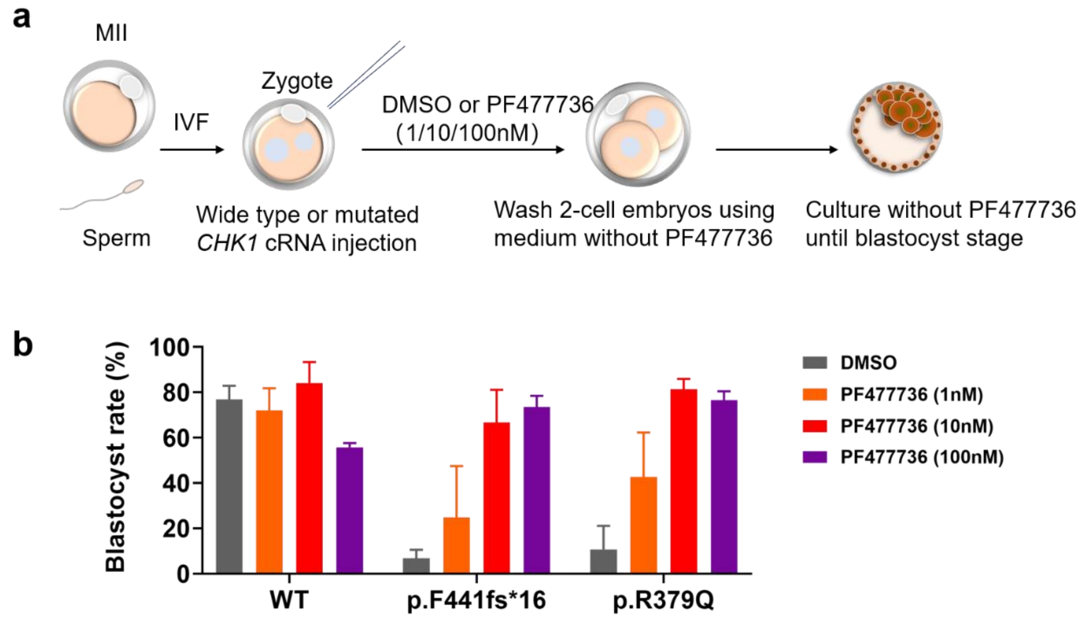

Figure S7: Optimizing the concentration of PF477736.

a A diagram depicting the flow of blastocyst culture. Mouse zygotes overexpressing the mutant (p. F441fs*16 or p.R379Q) or WT CHK1 were treated with PF477736 at different concentrations until the 2-cell embryo stage. Then the mouse 2-cell embryos were washed and transferred to fresh medium and were cultured to blastocyst stage. **b** Blastocyst rates of zygotes carrying WT or mutant hCHK1 under treatments of DMSO or PF477736 at different concentrations (1/10/100nM). PF477736 at the concentration of 10nM had the best rescuing efficiency in general.

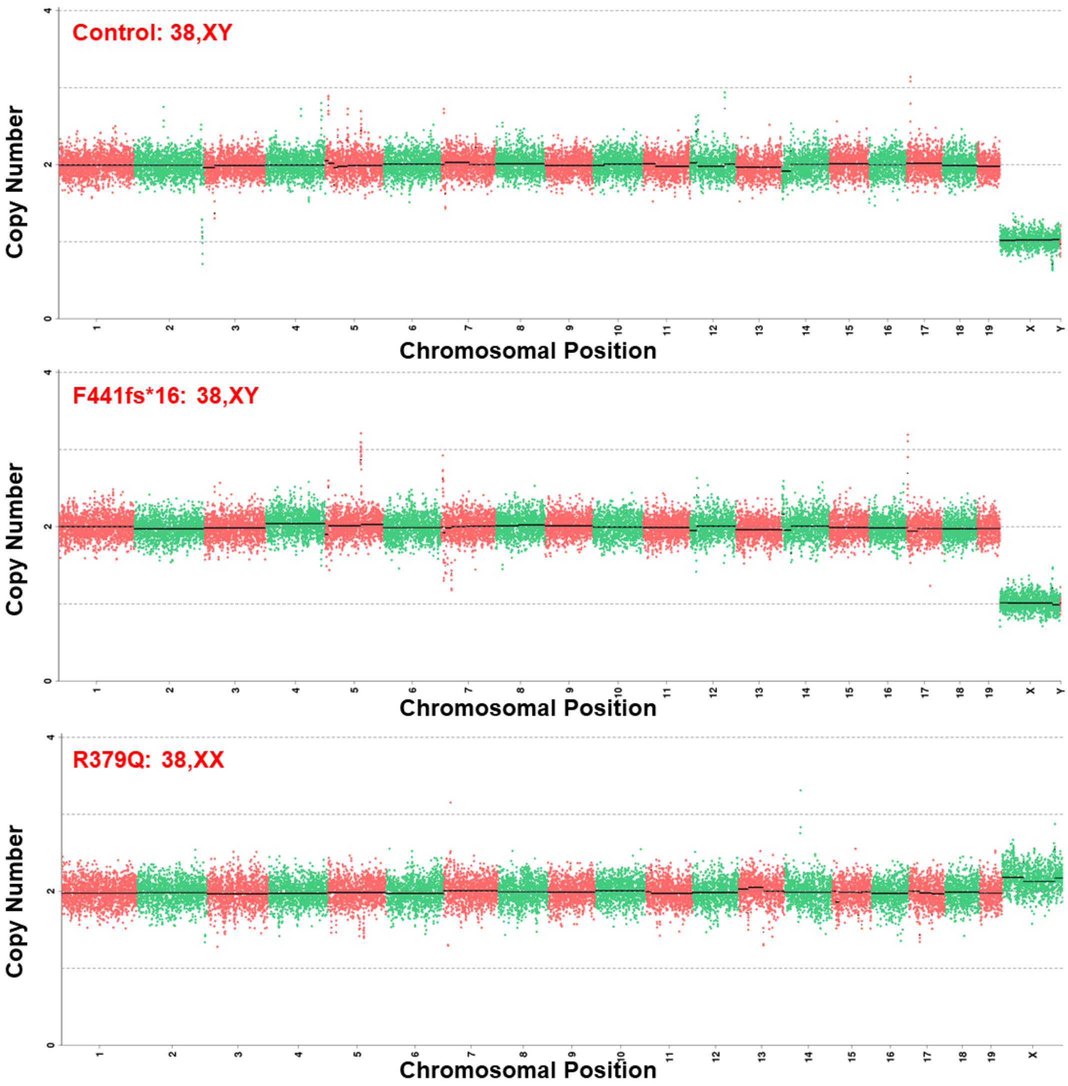

Figure S8: Representative CNV-seq images of mouse blastocysts treated with PF477736.

The blastocysts derived from control zygotes or zygotes overexpressing mutant CHK1 treated with PF477736 were collected individually for CNV-seq. The genome sequence of mutant blastocysts was aligned with the sequence of normal control blastocysts. No chromosome aneuploidy abnormalities or chromosomal deletions or duplications larger than 4Mb has been found in the mutant blastocysts treated with PF477736.

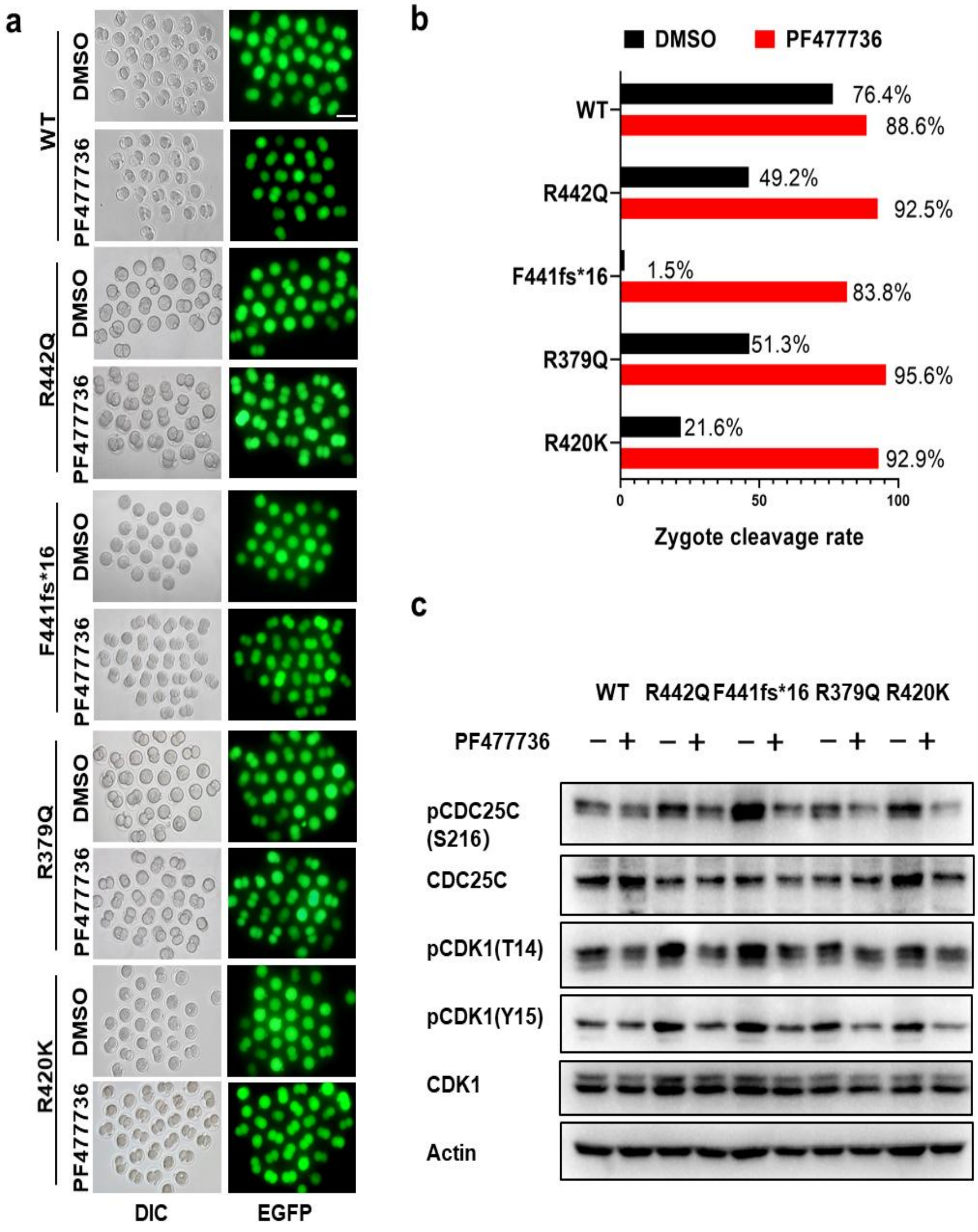

Figure S9. The CHK1 inhibitor can rescue mouse zygote arrest.

a-b Mouse zygotes injected with WT or mutant *CHK1* cRNAs were treated with DMSO or CHK1 inhibitor PF477736, respectively. **a** 18 hours later, typical images were exhibited. Scale bar:10μm. **b** The inhibitor PF477736 could significantly increase the mitosis rate of zygotes carrying these mutations ($p<0.05$). The Chi-square test was

used and a total of about 90 zygotes were calculated in each group in triplicates. On the right side is zygote cleavage rate of each group. **c** HEK-293T cells were treated with DMSO or PF477736 for another 18 hours after transfection for 30 hours. The western blot results indicated CHK1 inhibitor PF477736 could significantly reduce the expression of CHK1 downstream proteins (pCDC25C and pCDK1s).

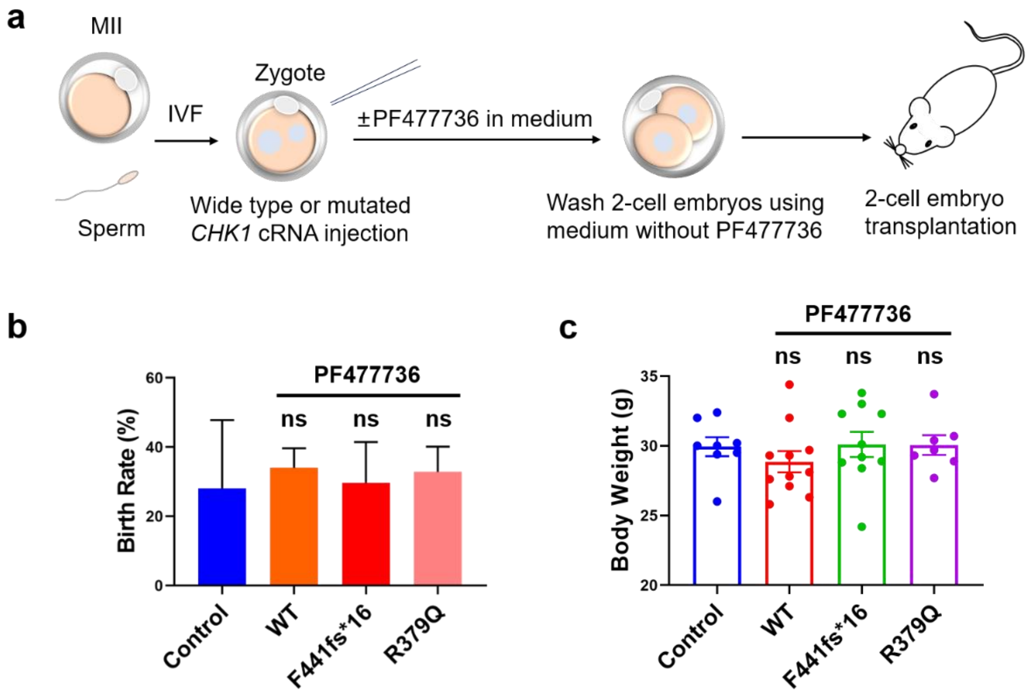

Figure S10: Mouse mutant zygotes can generate normal offspring under the treatment with PF477736.

a A diagram depicting the flow of embryo transfer. The mouse zygotes overexpressing the mutation p. F441fs*16 or p.R379Q were treated with PF477736 at 10nM until the 2-cell embryo stage. Then the mouse 2-cell embryos were transferred to pseudo-pregnant female mice in order to observe the litter. **b** There is no difference in birth rates between the normal control group without any treatment and WT or mutant groups treated with 10nM PF477736 based on a t-test. Bars indicate means ± SEM . **c** Weight of pups per group was documented 12 weeks after birth and there was no significant difference between the wild-type (WT) or mutated (F441fs*16/R379Q) groups treated with PF477736 and the normal control group (t-test). Bars indicate means ± SEM, ns: no significant difference.

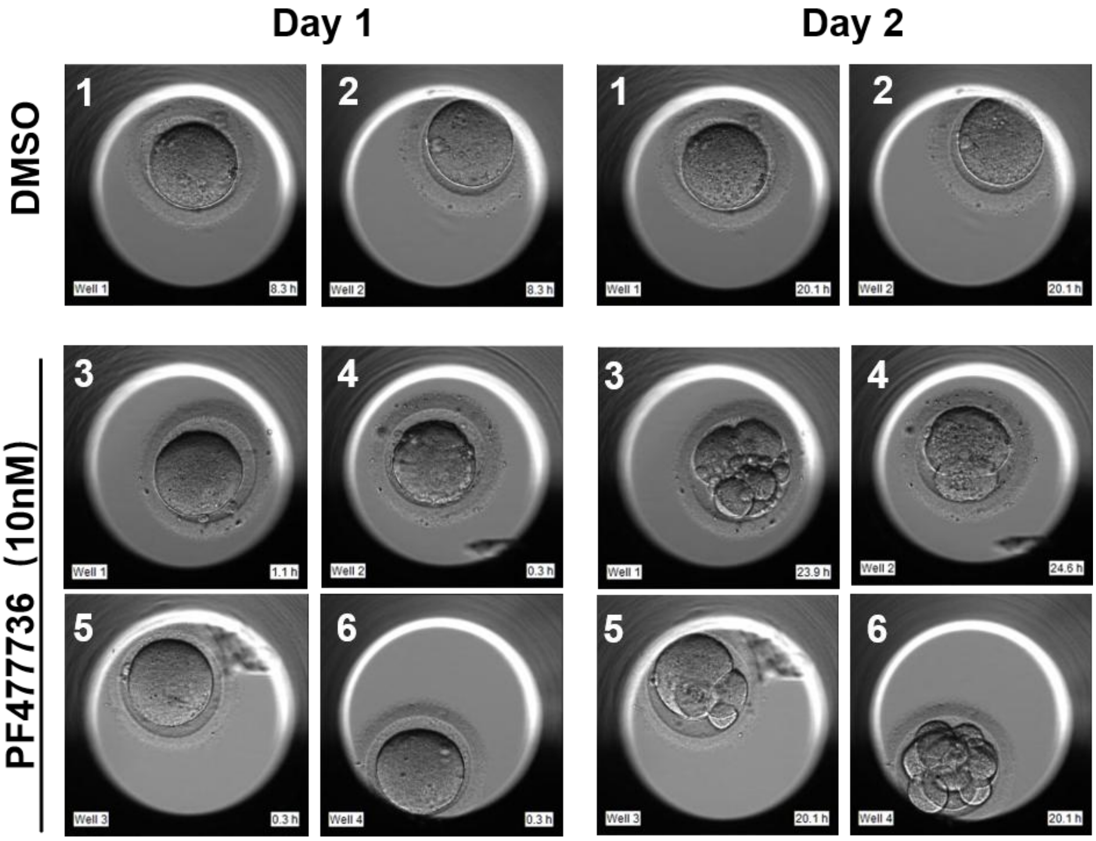

Figure S11. The blocked zygotes from patient III-2 (Family 1) could resume cleavage under the treatment with PF477736.

The patient's zygotes had been cultured until the third embryo day without cleavage and undergone cryopreservation for further research. Those zygotes were then treated with PF477736 (10nM) for another day after thawing, and they were able to divide and further develop. On the contrary, zygotes treated with DMSO were not able to divide. The white numeral indicates the number of each embryo.

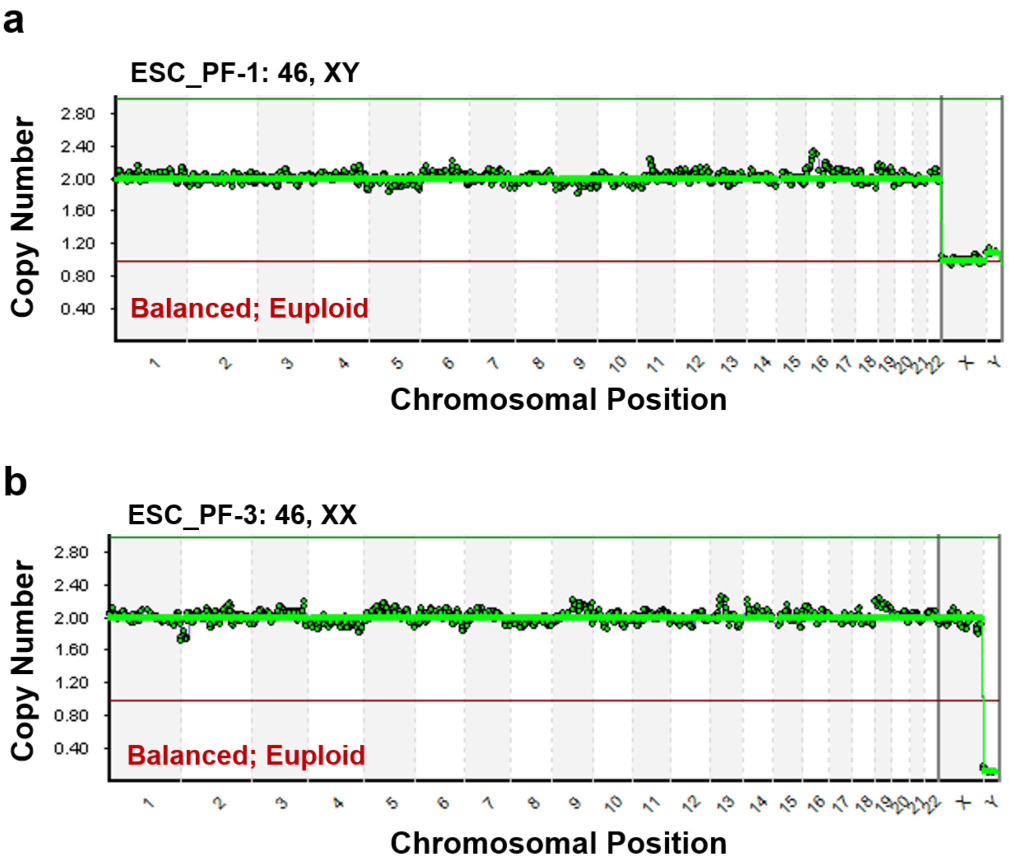

Figure S12. CNV-seq results of the two embryonic stem cell lines derived from patient (III-2, Family 1).

The two blastocysts, derived from the patient under the treatment with PF477736, were employed to establish embryonic stem cells, ESC_PF-1 (a) and ESC_PF-3 (b). The results reveal that both of two lines maintain the genome integrity.

Supplementary Tables

Table S1. Oocyte and embryo characteristics of IVF and ICSI cycles in the patients with *CHK1* mutations.

Patient	Mutation	Age (Years)	Duration of infertility (Years)	IVF/ICSI cycles	Retrieved oocytes	Mature oocyte	Fertilized oocytes	Fertilized oocytes states on Day 1	Embryos that could be transferred
III-2 in Family 1	R379Q	28	3.5	1- IVF	11	10	10	8 in PN, 2 in 1C	0
				2-ICSI	15	13	9	1 in PN, 8 in 1C	0
				3-ICSI	8	5	5	5 in PN	0
II-1 in Family 2	F441fs*16	31	7	1-IVF	13	12	11	11 in PN	0
				2-ICSI	12	9	8	6 in PN, 2 in 1C	0
				3-ICSI	7	7	6	6 in PN	0
II-2 in Family 3	R442Q	27	5	1-IVF	17	17	15	15 in PN	0
				2-ICSI	8	7	4	2 in PN, 2 in 1C	0
				3-ICSI	5	5	5	5 in PN	0
				4-ICSI	7	6	2	2 in PN	0
II-1 in Family 4	R420K	36	7	1-IVF	9	7	5	2 in PN, 3 in 1C	0
				2-ICSI	7	7	5	3 in PN, 2 in 1C	0
II-2 in Family 4	R420K	32	10	1-IVF	5	3	3	3 in PN	0
				2-IVF	8	8	8	8 in 1C	0

***** IVF denotes in vitro fertilization, ICSI intracytoplasmic sperm injection, Day 1 the first cleavage day, PN pronucleus, C cell.

Table S2: Overview of the *CHK1* mutations observed in the four families

Family	Pattern	cDNA change	Protein change	Mutation type	SIFT [a]	PPH2 [a]	Mutation Taster [a]	1000g_A [b]	gnomAD [b]	ACMG [c]
1	inherited	c.G1136A	p.R379Q	missense	D	D	D	NA	NA	LP
2	de novo	c.1323delC	p.F441fs*16	frameshift deletion	NA	NA	NA	NA	NA	LP
3	de novo	c.G1325A	p.R442Q	missense	D	D	D	NA	NA	LP
4	unknown	c.G1259A	p.R420K	missense	D	P	D	NA	NA	LP

Abbreviations are as follows: D, damaging; P, probably damaging; NA, not available；LP: Likely pathogenic

[a] Mutation assessment by SIFT, Polyphen-2 (PPH2) and Mutation Taster.

[b] Allele frequency of corresponding mutations in all population of 1000 Genomes (1000g_E) and gnomAD database.

[c] Mutation assessment according to the criteria of the American College of Medical Genetics and Genomics (ACMG).

Table S3: CNV-seq results of mouse blastocysts with mutations after treatment with PF477736

Blastocyst	CNV-seq result
F441fs*16-1	Balanced, Euploid
F441fs*16-2	Balanced, Euploid
F441fs*16-3	Balanced, Euploid
F441fs*16-4	Balanced, Euploid
R379Q-1	Balanced, Euploid
R379Q-2	Balanced, Euploid
R379Q-3	Balanced, Euploid
R379Q-4	Balanced, Euploid

Table S4: **Mouse embryo transfer record.**

Group	Zygote number	Transfer number	Recipient number	Pups	Birth rate
Control	100	50	2	7	14%
		50	2	21	42%
WT	100	50	2	19	38%
		50	2	15	30%
F441fs*16	97	47	2	10	21 %
		50	2	19	38%
R379Q	97	50	2	19	38%
		47	2	13	28%

Table S5: Embryo development record after treatment with CHK1 inhibitor PF477736.

	Control		Treated with PF477736				
**Em ID**	Ctrl-1	Ctrl-2	PF-1	PF-2	PF-3	PF-4	PF-5
**Zygote**	2PN	2PN	2PN	1PN	2PN	2PN	2PN
**Em-D1**	UD	UD	2C3'	2C3'	2C3'	1C	2C2'
**Em-D2**	UD	UD	4C4'	2C3'	4C4'	4C3'	2C2'
**Em-D3**	UD	UD	8C3'	8C2'	8C3'	6C2'	4C2'
**Em-D5/6**	UD	UD	4BB	D	4AA	D	D

"Em" represents embryo; "D" represents day; "UD" represents undivided; "D" represents degenerated

Table S6: Primers used for *CHK1* exon sequencing

**Primers for exon sequencing**	**Sequence**
CHK1-Exon2-F	GCTGTTAATTTTCGTGGGCA
CHK1-Exon2-R	TTCAGTTGCCAAAACCCTTG
CHK1-Exon3-F	TGAGAACATAGCAGAAACCACT
CHK1-Exon3-R	TCCAATTTCACAGTTGCATGAG
CHK1-Exon4_5-F	AAGCCCCATATGTGTTAGTGG
CHK1-Exon4_5-R	AGACTTGATTTTGCCTTGTATGG
CHK1-Exon6-F	TGATGAGGGGCCTTGCTTTA
CHK1-Exon6-R	TCTGGCCAAGAGTGAGACC
CHK1-Exon7-F	TGAAGTGCCTCTAAAGTTTCCA
CHK1-Exon7-R	TGCTCTGAATATACACTCCCCA
CHK1-Exon8-F	ACTCCAAGATACAGCAGCAGA
CHK1-Exon8-R	GCTATCATGTGTTGTTGACTTGT
CHK1-Exon9-F	ACTCCACACTTTGAACATGTCT
CHK1-Exon9-R	TCACACACAAGTTCTCATGCT
CHK1-Exon10-F	TCAGGTGGTGTGTCAGAGTC
CHK1-Exon10-R	GCCTCCCTCCTCTCTTTCTT
CHK1-Exon11-F	GGGAGGCCTTCATGCAAAAT
CHK1-Exon11-R	CACCCCAGCCTCCCAAAA
CHK1-Exon12-F	CCTGGTCTGAAGCGATCCT
CHK1-Exon12-R	AGTCTCTTGTATTGTCACCCAGA
CHK1-Exon13-F	AGGAACAGTGATGGGCATGA
CHK1-Exon13-R	TGGATAAACAGGGAAGTGAACAC

Table S7: Primers used for site-directed mutagenesis and qRT-PCR

**Primers used for site-directed mutagenesis**	**Sequence**
CHK1-LF097-F	GGTTGGTCAAAAGAATGACACAATTCTTTACC AAATTGGATGC
CHK1-LF097-R	GCATCCAATTTGGTAAAGAATTGTGTCATTCTT TTGACCAACC
CHK1-LF057-F	AGAAATGGATGATAAAATATTGGTTGACTTCGG CTTTCTAAGGTATTTT
CHK1-LF057-R	AAAATACCTTAGAAAGCCGAAGTCAACCAATAT TTTATCATCCATTTCT
CHK1-LF033-F	GGATGATAAAATATTGGTTGACTTCCAGCTTTC TAAGGTATTTTTATGTTTTA
CHK1-LF033-R	TAAAACATAAAAATACCTTAGAAAGCTGGAAGT CAACCAATATTTTATCATCC
CHK1-LF160-F	GGTTACTATATCAACAACTGATAGGAAAAACAA TAAACTCATTTTCAAAGTGA
CHK1-LF160-R	TCACTTTGAAAATGAGTTTATTGTTTTTCCTATC AGTTGTTGATATAGTAACC
CDC25C-F	AGTTCTCTGGCATCGCCGGGGAGCGATATAG
CDC25C-R	CTATATCGCTCCCCGGCGATGCCAGAGAACT
CDK1-F	CCCTTATACACAACTCCAGCGGCACCTTCTCC AATTTTCTCTATTTTGGTATAATCTT
CDK1-R	AAGATTATACCAAAATAGAGAAAATTGGAGAAG GTGCCGCTGGAGTTGTGTATAAGGG
**Primers used for qRT-PCR**	**Sequence**
CHK1-RT-F	GATCATCAGCAATGCCTCCT
CHK1-RT-R	TTCAGCTCAGGGATGACCTT
GAPDH-RT-F	CTGCACCACCAACTGCTTAG
GAPDH-RT-R	GGATGCAGGGATGATGTTCT

Table S8: List of antibodies used in immunofluorescence and western blot

Antibodies	Source	Identifier
OCT4 Antibody	Santa Cruz	sc-5279
Sox2 (D6D9) Antibody	Cell Signaling Technology	3579S
SSEA4 Antibody	Abcam	ab16287
TRA-1-60 Antibody	Abcam	ab16288
TRA-1-81 Antibody	Santa Cruz	sc-21706
Beta Actin Monoclonal Antibody	Protein tech	66009-1-Ig
GAPDH Monoclonal Antibody	Protein tech	60004-1-Ig
Anti-CDK1	Abcam	ab32094
Anti-CDK1 (phospho T14)	Abcam	ab58509
Phospho-cdc25C (Ser216) Antibody	Cell Signaling Technology	9528S
Anti-Cdc25C	Abcam	ab226958
Anti-p-Cdc2 p34 (pY15.44)	Santa Cruz	sc-136014

Supplementary References

1. Zhang X, Wu K, Zheng Y, et al. In vitro expansion of human sperm through nuclear transfer. Cell Res. 2020; 30:356–359.
2. Yan L, Yang M, Guo H, et al. Single-cell RNA-Seq profiling of human preimplantation embryos and embryonic stem cells. Nat Struct Mol Biol 2013; 20:1131-1139.
3. Wang J, Han X, Feng X, Wang Z, Zhang Y. Coupling cellular localization and function of checkpoint kinase 1 in checkpoints and cell viability. J Biol Chem 2012; 287:25501-25509.

Web Resources

OMIM, https://www.omim.org/

GeneBank, https://www.ncbi.nlm.nih.gov/genbank/

Swiss Model web server, https://swissmodel.expasy.org/

PyMol software, https://pymol.org/2/

Clustalx software, http://www.clustal.org/download/current/

Genome Aggregation Database (gnomAD), https://gnomad.broadinstitute.org/

1000 Genomes Project, https://www.internationalgenome.org/home

dbSNP, https://www.ncbi.nlm.nih.gov/projects/SNP/

SIFT, https://sift.bii.a-star.edu.sg/

PolyPhen-2, http://genetics.bwh.harvard.edu/pph2/

MutationTaster, http://mutationtaster.org/MutationTaster/
